# An outbreak of foodborne norovirus gastroenteritis linked to a restaurant in Melbourne, Australia, 2014

**DOI:** 10.5365/WPSAR.2017.8.1.008

**Published:** 2017-05-05

**Authors:** Shaun P. Coutts, Kaye Sturge, Karin Lalor, John A. Marshall, Leesa D. Bruggink, Nela Subasinghe, Marion Easton

**Affiliations:** a Victorian Department of Health and Human Services, Melbourne, Australia.; b Victorian Infectious Diseases Reference Laboratory, Melbourne, Australia.; c Microbiological Diagnostic Unit Public Health Laboratory, Melbourne, Australia

## Abstract

**Introduction:**

In May 2014 an outbreak of norovirus occurred among patrons of a restaurant in Melbourne, Australia. Investigations were conducted to identify the infectious agent, mode of transmission and source of illness, and to implement controls to prevent further transmission.

**Methods:**

A retrospective case-control study was conducted to test the hypothesis that food served at the restaurant between 9 and 15 May 2014 was the vehicle for infection. A structured questionnaire was used to collect demographic, illness and food exposure data from study participants. To ascertain whether any food handlers had experienced gastroenteritis symptoms and were a possible source of infection, investigators contacted and interviewed staff who had worked at the restaurant between 9 and 16 May 2014.

**Results:**

Forty-six cases (including 16 laboratory-confirmed cases of norovirus) and 49 controls were interviewed and enrolled in the study. Results of the analysis revealed a statistically significant association with illness and consumption of grain salad (OR: 21.6, 95% CI: 1.8–252.7, *P* = 0.015) and beetroot dip (OR: 22.4, 95% CI: 1.9–267.0, *P* = 0.014). An interviewed staff member who reported an onset of acute gastrointestinal illness on 12 May 2014 had prepared salads on the day of onset and the previous two days.

**Discussion:**

The outbreak was likely caused by person-to-food-to-person transmission. The outbreak emphasizes the importance of the exclusion of symptomatic food handlers and strict hand hygiene practices in the food service industry to prevent contamination of ready-to-eat foods and the kitchen environment.

## Introduction

Noroviruses are non-enveloped, single-stranded RNA viruses, recognized as a leading cause of acute gastroenteritis worldwide. (*1*) There are currently six recognized norovirus genogroups, three of which (GI, GII and GIV) cause human illness. (*2*) Norovirus is transmitted via the faecal–oral route primarily through close contact with an infected person, contact with contaminated fomites or consumption of contaminated food or water. (*3*) The average incubation period is between 24 and 48 hours, with symptoms including acute-onset vomiting, diarrhoea, nausea, myalgia and low-grade fever. (*4*) Infected individuals shed the virus while symptomatic; however, shedding has been documented before the onset of symptoms, after symptoms have resolved and by asymptomatic infected individuals. (*5*-*7*) Contamination of food by both symptomatic and asymptomatic infected food handlers has been well documented. (*8*-*14*)

Commencing 13 May 2014, the Victorian Department of Health and Human Services, Communicable Disease Prevention and Control Unit received reports of gastrointestinal illness in patrons following a banquet lunch at a Mediterranean-style restaurant on 11 May 2014. In response, an outbreak investigation was initiated with the local council health department to identify the infectious agent, the mode of transmission, the source of illness and to implement controls to prevent further transmission.

## Methods

### Epidemiological investigation

A retrospective case-control study was conducted to test the hypothesis that food served at the restaurant was the vehicle for infection. Study participants were recruited from the restaurant’s booking list and contacted for phone interviews. A structured questionnaire was used to collect demographic, illness and food exposure data from study participants.

A probable case was defined as a person who ate food at the restaurant between 9 and 15 May 2014 and had onset of vomiting and/or diarrhoea or two or more symptoms of fever, nausea, abdominal pain and headache between 24 and 48 hours after consumption. A confirmed case met the probable case definition and also had norovirus detected by polymerase chain reaction (PCR) in a faecal specimen. Controls were patrons identified during the interview process who did not meet the definition of a probable or confirmed case but had eaten at the restaurant between 9 and 15 May 2014.

To ascertain whether any food handlers had experienced gastroenteritis symptoms and were a possible source of infection, investigators contacted and interviewed staff who worked at the restaurant between  9 and 16 May 2014.

Data analysis was conducted using Stata 13 (StataCorp, College Station, TX). Univariate analysis was used to calculate *p*-values (2-sided Fisher exact), odds ratios (OR) and 95% confidence intervals (CI) for food exposure variables. Variables with a *p*-value < 0.05 in univariate analysis were included in a multivariable logistic regression model. Backward elimination was used to refine the model with the variable with the highest *p*-value > 0.05 removed at each elimination step. Variables found to be statistically significant on univariate and multivariable analyses were reported.

### Environmental and laboratory investigation

Environmental health officers from the council health department conducted an environmental investigation at the restaurant. Food samples were obtained and tested for *Salmonella* spp., coagulase-positive staphylococci, *Bacillus cereus* and *Clostridium perfringens* at the Microbiological Diagnostic Unit Public Health Laboratory (MDU PHL). Food samples were not tested for norovirus as the molecular detection of norovirus in food remains prohibitively expensive, time consuming and often unsuccessful because of the heterogeneous distribution of low numbers of virus particles in foods. (*13*)

Stool specimens were obtained from cases where possible and tested for bacterial enteric pathogens at MDU PHL. The specimens were tested for norovirus by reverse transcription polymerase chain reaction (RT–PCR) at the Victorian Infectious Diseases Reference Laboratory and nucleotide sequencing of norovirus RNA was conducted where appropriate, as previously reported. (*14*)

### Ethics and permissions

Ethics approval was not sought as the investigation was undertaken as part of a public health response to an outbreak.

## Results

### Epidemiological investigation

Forty-six cases (including 16 confirmed cases) and 49 controls were identified and interviewed. The majority of cases dined at the restaurant on 11 May 2014 with most experiencing an onset of symptoms during the afternoon of 12 May 2014 or the morning of 13 May 2014. The last recorded case ate at the restaurant on 15 May 2014 and had an onset of symptoms on 17 May 2014. An incubation period was recorded for 29 cases, and a median of 28 hours (range 7 to 57 hours) was observed. Symptom duration was recorded for 27 cases, and a median duration of two days (range 0.5 to 5 days) was reported. Symptom characteristics of the cases are described in **Table 1**.

**Table 1:** Symptom characteristics of 46 cases identified among patrons of a restaurant in Melbourne,  Australia, 2014

Symptom	Number of cases reporting	Percentage of cases reporting
Diarrhoea	45	98%
Abdominal pain	39	85%
Nausea	33	72%
Vomiting	29	63%
Headache	27	59%
Fever	16	35%

Twelve staff members who worked between 9 and 16 May were interviewed during the investigation. Only one staff member reported experiencing acute gastroenteritis during this period with an onset of illness on 12 May 2014. The staff member had prepared salads on the day of onset and the previous two days. The staff member was unwilling to provide a faecal specimen for laboratory testing.

In univariate analysis, foods significantly associated with illness were the beetroot dip, grain salad (a cold salad containing freekeh wheat, lentils, parsley and nuts), coleslaw, calamari and lamb cutlets (**Table 2**). In the final multivariable logistic regression model, two foods remained significantly associated with illness: the grain salad (OR: 21.6, 95% CI: 1.8–252.7, *P* = 0.015) and the beetroot dip (OR: 22.4, 95% CI: 1.9–267.0, *P* = 0.014).

**Table 2:** Food items associated with illness by univariate analysis

Food Item	Cases (*n* = 46) (%)	Controls (*n* = 49) (%)	Odds ratio (95% confidence interval)		*p*-value
Beetroot dip	11 (24)	1 (2)	15.1	(2.0–662.4)	0.001
Grain salad	45 (98)	37 (76)	14.6	(2.0–637.3)	0.002
Coleslaw	18 (39)	9 (18)	2.9	(1.0–8.3)	0.025
Calamari	9 (20)	2 (4)	5.7	(1.1–56.6)	0.018
Lamb cutlets	9 (20)	2 (4)	5.7	(1.1–56.6)	0.018

A review of food frequency data revealed 45 cases (98%) recalled eating the grain salad, and one case did not consume any other food from the restaurant except the grain salad. By comparison, only 11 of the 46 cases (24%) reported consumption of the beetroot dip.

### Environmental and laboratory investigation

Sixteen of the 23 stool specimens were positive for GII norovirus. Additional studies indicated this was the epidemic strain GII.Pe/GII.4_Sydney_2012. All food and stool samples tested were negative for bacterial pathogens.

A senior staff member at the restaurant reported the grain salad was generally prepared in batches of approximately 12 kg to be used over a period of three to four days. It was mixed in large containers by hand; gloves were not always worn during the mixing process. An unusually large 19 kg batch of grain salad was made in preparation for the 11 May 2014 lunch. The salad did not undergo any processing steps (such as cooking) that would have inactivated norovirus potentially introduced by an infected food handler during preparation.

The environmental investigation revealed some of the handwashing facilities in food preparation areas were obstructed and not supplied with soap.

## Discussion

This point source outbreak of norovirus among restaurant patrons is likely to have been caused by person-to-food-to-person transmission. The results of the case-control study analysis, the higher frequency of consumption of the grain salad among cases, the likelihood an infectious food handler prepared this menu item and the ready-to-eat nature of the product all support the grain salad as the most likely vehicle of infection, though it is possible that the beetroot dip may have also been contaminated. The large batch size of the grain salad and the extended time over which it was served likely contributed to the protracted onsets of illness among patrons.

Although norovirus infection was not confirmed in any food handlers at the restaurant, it is suspected that ready-to-eat food was contaminated by a food handler during the pre-symptomatic or the early-symptomatic stage of illness. The lack of adequate handwashing facilities in food preparation areas further supports this hypothesis, as does anecdotal evidence that salads were often mixed with bare hands. National food standards in Australia require food handlers to take all practical measures to prevent unnecessary contact with ready-to-eat food and detail requirements for the availability and use of handwashing facilities by food handlers. (*15*)

National food standards in Australia also require a food business to exclude employees having a foodborne disease from handling food until a medical practitioner advises the employee no longer has or is carrying the disease. (*15*) While this requirement aims to minimize the risk of food contamination by ill food handlers, it may also discourage reporting of gastroenteritis among food handlers, many of whom are employed on a casual basis and receive no entitlements when absent from work for medical reasons.

Several limitations were identified in this study. The retrospective nature of the case-control study makes it impossible to rule out recall bias, which may have been exacerbated by the large number of food choices available on the banquet menu. The lack of a practical method for the molecular detection of norovirus in food samples meant that it was not possible to confirm the presence of norovirus in the implicated foods. Food handlers, including asymptomatic individuals, were not tested for norovirus infection during the investigation, so the presence of an infected food handler at the premises could not be laboratory-confirmed.

This outbreak emphasizes the importance of the exclusion of symptomatic food handlers and strict hand hygiene practices in the food service industry to prevent contamination of ready-to-eat foods and the kitchen environment. It is essential that food regulators continue to promote and enforce these requirements on food business operators to prevent future outbreaks of norovirus caused by infectious food handlers.
